# Hepatoprotective Effects of a Natural Flavanol 3,3′-Diindolylmethane against CCl_4_-Induced Chronic Liver Injury in Mice and TGFβ1-Induced EMT in Mouse Hepatocytes via Activation of Nrf2 Cascade

**DOI:** 10.3390/ijms231911407

**Published:** 2022-09-27

**Authors:** Suvesh Munakarmi, Yamuna Gurau, Juna Shrestha, Prabodh Risal, Ho Sung Park, Hyun Beak Shin, Yeon Jun Jeong

**Affiliations:** 1 Research Institute of Clinical Medicine of Jeonbuk National University, Biomedical Research Institute, Jeonbuk National University Hospital, Jeonju 54907, Korea; 2 Alka Hospital Private Limited, Jwalakhel, Kathmandu 446010, Nepal; 3 Department of Biochemistry, School of Medical Sciences, Kathmandu University, Dhulikhel 45200, Nepal; 4 Department of Pathology, Jeonbuk National University Hospital, Jeonju 54907, Korea; 5 Department of Surgery, Jeonbuk National University Hospital, Jeonju 54907, Korea

**Keywords:** DIM, CCl_4_, TGFβ1, EMT, liver fibrosis, oxidative stress, inflammation, ROS

## Abstract

Hepatic fibrosis is a form of irregular wound-healing response with acute and chronic injury triggered by the deposition of excessive extracellular matrix. Epithelial–mesenchymal transition (EMT) is a dynamic process that plays a crucial role in the fibrogenic response and pathogenesis of liver fibrosis. In the present study, we postulated a protective role of 3,3′-diindolylmethane (DIM) against TGF-β1 mediated epithelial–mesenchymal transition (EMT) in vitro and carbon tetrachloride (CCl4)-induced liver fibrosis in mice. TGF-β1-induced AML-12 hepatocyte injury was evaluated by monitoring cell morphology, measuring reactive oxygen species (ROS) and mitochondrial membrane potential, and quantifying apoptosis, inflammatory, and EMT-related proteins. Furthermore, CCl4-induced liver fibrosis in mice was evaluated by performing liver function tests, including serum ALT and AST, total bilirubin, and albumin to assess liver injury and by performing H&E and Sirius red staining to determine the degree of liver fibrosis. Immunoblotting was performed to determine the expression levels of inflammation, apoptosis, and Nrf2/HO-1 signaling-related proteins. DIM treatment significantly restored TGF-β1-induced morphological changes, inhibited the expression of mesenchymal markers by activating E-cadherin, decreased mitochondrial membrane potential, reduced ROS intensity, and upregulated levels of Nrf2-responsive antioxidant genes. In the mouse model of CCl4-induced liver fibrosis, DIM remarkably attenuated liver injury and liver fibrosis, as reflected by the reduced ALT and AST parameters with increased serum Alb activity and fewer lesions in H&E staining. It also mitigated the fibrosis area in Sirius red and Masson staining. Taken together, our results suggest a possible molecular mechanism of DIM by suppressing TGF-β1-induced EMT in mouse hepatocytes and CCl4-induced liver fibrosis in mice.

## 1. Introduction

The burden of liver disease is rising rapidly worldwide due to a distinct regenerative response of the liver to injuries. Liver injury can be classified as acute, chronic, cholestatic, and mixed patterns of injury depending on its multiple causes, including toxins, viral hepatitis, non-alcoholic steatohepatitis (NASH), and alcoholism [1,2]. However, long-time exposure to a toxic agent can cause irreversible liver damage, parenchymal changes, and vascular architectural disruption, which can gradually lead to liver fibrosis, cirrhosis, and finally, hepatocellular carcinoma (HCC), which is the end-stage of most chronic liver diseases [3,4,5]. Although numerous pathogenic factors, including lipotoxicity, oxidative stress, and mitochondrial dysfunction, influence the transition from simple steatosis to NASH, the molecular mechanism for NASH progression remains unclear due to which sensitive, non-invasive diagnostic methods for NASH are absent, thus impeding the development of therapeutically relevant animal models and pharmaceutical therapies for NASH [6,7].

Liver fibrosis is a complex pathophysiological process characterized by an excessive deposition of extracellular matrix (ECM) α-smooth muscle actin (α-SMA) proteins that are responsible for chronic liver damage [8]. Transforming growth factor-β (TGF-β) is the type of profibrogenic cytokine that can promote tumor invasion and metastasis by inducing EMT as well as promoting inflammatory responses by activating interleukin-1 (IL-1β) and interleukin-6 (IL-6) in monocytes [9]. Numerous studies have suggested that TGF-β1 signaling plays a pivotal role in the development and progression of liver fibrosis [10]. EMT is the process of transforming hepatocytes into fibroblasts to develop liver fibrosis [11]. EMT can interrupt intracellular junctions of epithelial cells with the downregulation of E-cadherin and the tight junctions protein Zonula Ooccludens (ZO-1) and the upregulation of mesenchymal markers such as N-cadherin, fibronectin, vimentin, and snail [12]. Carbon tetrachloride (CCl_4_) is an extensively used xenobiotic in labs to make injury models. CCl_4_-induced liver fibrosis is associated with the accumulation of lipid-derived oxidation due to lipid peroxidation from free radicals and the diminution of antioxidant status [13]. Toxic substances produced from lipid peroxidation can lead to mitochondrial DNA damage that causes the destruction of normal cellular structure with the loss of nuclei and amounts of fragmental and condensed nucleus [14]. Therefore, exploring the mechanism of EMT and mitochondrial-dependent apoptotic cell death, blocking TGF-β production, blocking signal transduction, and/or inhibiting oxidative stress could provide promising therapeutic strategies to control and even reverse liver fibrosis.

Mitochondrial damage and oxidative stress are hallmarks of several diseases, including clinical and experimental liver diseases. During energy production in a cell, mitochondria play a crucial role in a variety of functions such as apoptosis, calcium homeostasis, cell proliferation, metabolic substrate synthesis, and inflammation [15]. The overproduction of reactive oxygen species (ROS) can trigger mitochondrial DNA to induce oxidative stress, which reduces the expression of the proteins necessary for the electron transport chain, resulting in organelle dysfunction and eventually inducing cell death [16,17]. Oxidative stress also plays an important role in the production of chronic inflammation, which is common pathogenesis involved in the progression of liver injury [18,19]. Therefore, antioxidant and anti-inflammatory treatment has a beneficial effect in preventing liver injury by reducing mitochondrial damage.

In the pathogenesis of various diseases, cells have strongly evolved in the activation of endogenous antioxidant defense mechanisms against oxidative stress [20]. Transcription nuclear factor erythroid 2-related factor 2 (Nrf2) and antioxidant/electrophile response elements (ARE/EpRE)-regulated phase II detoxifying enzymes genes such as hemeoxygenase-1 (HO-1), NADP(H): quinune oxidoreductase-1 (NQO1) are major antioxidant molecules involved in maintaining increased oxidative stress and balancing redox status in many tissues [21,22]. Antioxidant proteins such as glutathione peroxidase-4 (GPx-4) can inhibit phospholipid oxidation and ROS production in ferroptosis by acting as glutathione peroxidases [23,24]. Numerous studies have reported that the Nrf2/HO-1 antioxidant pathway is an effective therapeutic target for protection against liver diseases [25,26].

The treatment of liver disease with conventional and synthetic drugs has been controversial due to poor management and serious side effects. Therefore, it is necessary to discover traditional and herbal medicine with potential therapeutic effects on liver disease. In this study, we used 3,3′-diindolylmethane (DIM), a plant-derived natural flavanol extracted from cruciferous vegetables such as broccoli, Brussels sprout, cabbage, cauliflower, and kale. DIM is believed to possess anti-inflammatory, antioxidant, and anti-cancer properties, as stated in previous studies [27,28]. Previously, we have shown that DIM can effectively alleviate CCl_4_-induced acute liver injury in mice [27]. However, the effect of DIM has not been studied in liver fibrosis or in EMT. Moreover, the detailed cellular mechanism of DIM and its protective effect against liver injury remain unclear. Thus, the objective of this study was to explore whether DIM could be used as a promising therapeutic agent against CCl_4_-induced liver fibrosis. In addition, its mechanisms of action were explored by determining whether it could reduce lipid peroxidation, enhance hepatic glutathione content, and suppress TGF-β1 mediated EMT and apoptosis in mouse hepatocytes.

## 2. Results

### 2.1. DIM Ameliorates TGF-β1 Induced Hepatotoxicity in AML12 Cells

**Figure 1:**
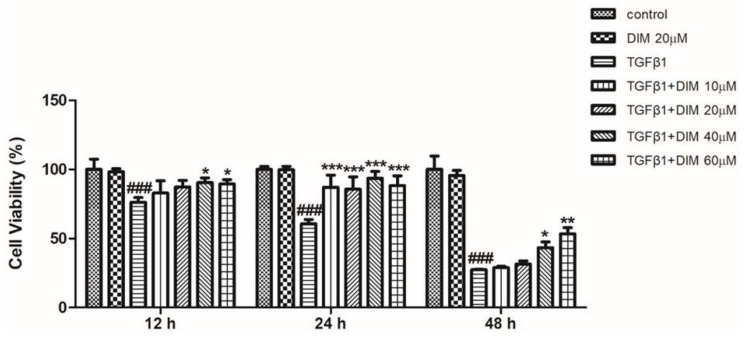
DIM inhibits TGF-β1 induced hepatocytes toxicity: MTT assay reveals that DIM could restore cell viability and reduce TGF-β1 induced cell toxicity of AML12 cells in both time- and concentration-dependent manner as compared with treatment with TGF-β1 only. Data are presented as mean ± SE from three identical experiments. ^###^, *p* < 0.001 denotes significant differences compared to the control; *, *p* < 0.05; **, *p* < 0.01; and ***, *p* < 0.001 compared to treatment with TGF-β1 only.

We performed an MTT assay to determine the effect of DIM against TGF-β1-induced cell toxicity in AML12 cells. As shown in Figure 1 the DIM treatment significantly increased the percentage of viable cells in a time and dose-dependent manner compared with treatment with TGF-β1 only.

### 2.2. DIM Treatment Restores TGF-β1-Induced Mesenchymal Phenotype and Inhibits EMT in AML12 Cells

**Figure 2:**
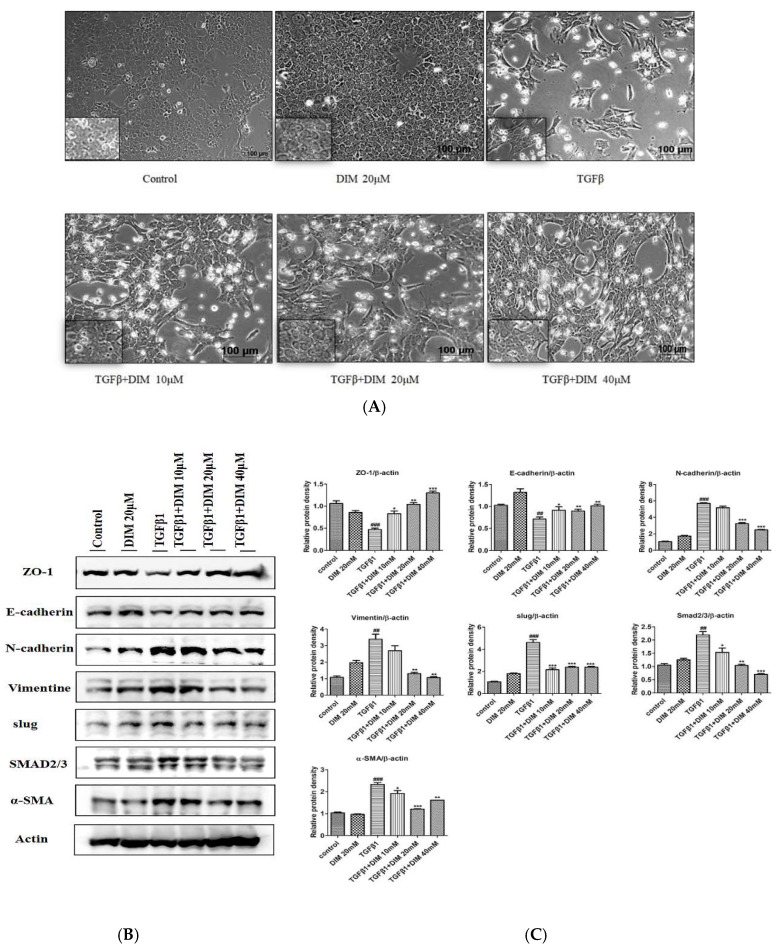
DIM inhibits mesenchymal phenotype, and EMT levels in AML-12 cells: (**A**) Pictures captured at 100× and 200× magnifications under a phase contrast microscope. (**C**) Western blot analysis determining the effect of DIM on protein expression of EMT-associated markers at 24 h after TGF-β1 treatment. (**B**) Quantitative analysis of relative protein expression normalized against β-actin. Data are presented as mean ± SE from three separate experiments (*n* = 3). ^##^, *p* < 0.01 and ^###^, *p* < 0.001 denote significant differences compared to the control; * *p* < 0.05, ** *p* < 0.01, and *** *p* < 0.001 compared to treatment with TGF-β1 only.

The mesenchymal phenotype induced by the TGF-β1 treatment was assessed by observing cell morphology under a phase-contrast microscope at X100 and X400 magnifications. As shown in Figure 2A, the DIM treatment suppressed TGF-β1-induced mesenchymal properties by restoring a honeycomb and spindle-like morphology to a polygonal morphology with a tight arrangement in comparison to the treatment with TGF-β1 only. Furthermore, we performed Western blotting to determine the effect of DIM on the expression of mesenchymal and epithelial markers. As shown in Figure 2B, DIM effectively restored levels of epithelial markers E-cadherin and ZO-1, whereas it reduced expression levels of mesenchymal markers such as N-cadherin, vimentin, slug, and SMAD2/3 compared with treatment with TGF-β1 only.

### 2.3. DIM Treatment Mitigates TGF-β-Induced ROS and Mitochondrial Dysfunction in AML12 Cells

**Figure 3:**
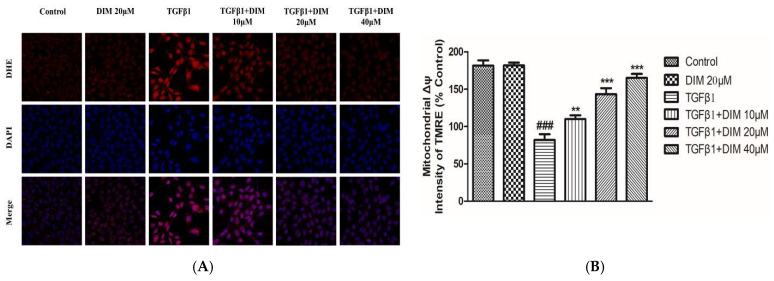
DIM suppresses hepatocyte injury by reducing intracellular ROS and improving mitochondrial membrane potential. (**A**) Intracellular ROS levels in AML-12 cells after TGF-β1 treatment for 24 h were evaluated by incubating cells with dihydroethidium (DHE), an ROS fluorescent probe, and monitoring under a confocal microscope. Scale bar, 30 µm. (**B**) Changes in mitochondrial membrane potential (ΔΨm) of AML-12 cells were determined after TGF-β1 treatment by staining cells with tetramethylrhodamine ethyl ester perchlorate (TMRE). All data are expressed as mean ± SE of three separate experiments (*n* = 3), ^###^ *p* < 0.001 denote significant differences compared to the control; **, *p* < 0.01, and ***, *p* < 0.001 compared to treatment with TGF-β1 only.

Reactive oxygen species (ROS) are considered important factors of cellular oxidative metabolism that play a crucial role in the regulation of cell survival, cell death, differentiation, cell signaling, and inflammatory response [29]. Similarly, mitochondria play a key role in the regulation of cellular redox homeostasis by serving as a powerhouse for cells. TGF-β1-induced hepatocytes apoptosis is closely related to the overproduction of reactive oxygen species (ROS), loss in mitochondrial membrane potential (ΔΨm), and subsequent mitochondrial programmed apoptosis [30]. To evaluate the effects of DIM, the intensity of ROS production and the loss of mitochondrial membrane potential were calculated. As shown in Figure 3A,B, the DIM treatment significantly inhibited fluorescence intensity and restored levels of mitochondrial membrane potential as compared to the treatment with TGF-β1 only, suggesting that DIM could mitigate TGF-β1-induced hepatocytes injury by inhibiting ROS production and damage in mitochondrial membrane potential.

### 2.4. DIM Suppresses TGF-β1-Induced Apoptosis in AML12 Cells

**Figure 4:**
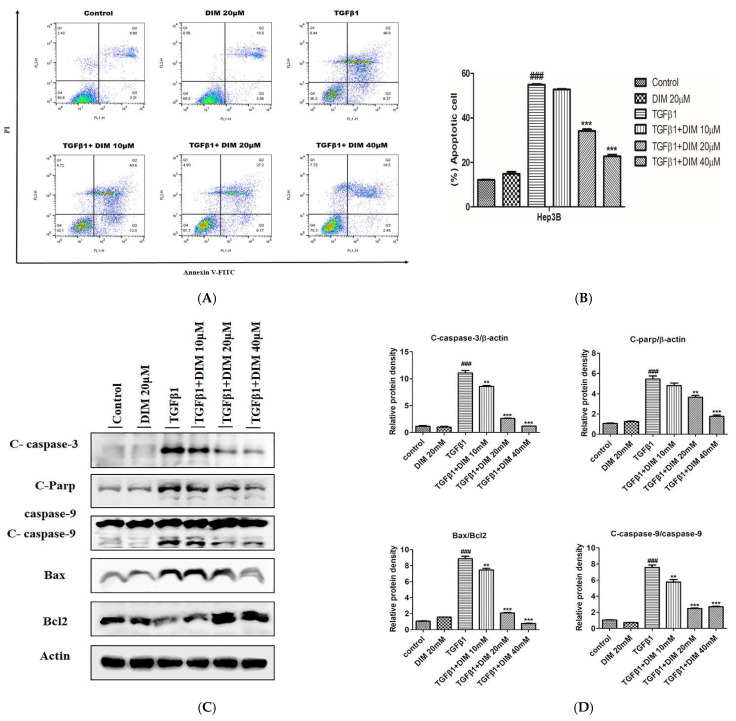
DIM pretreatment attenuates TGF-β1-induced AML-12 cell death. (**A**) FACS analysis using Annexiin V/PI staining determines the effect of DIM against TGF-β1-induced apoptosis of hepatocytes. (**B**) Quantitative assessment (%) of apoptotic early and late apoptotic cells. (**C**) Western blot analysis was used to evaluate levels of apoptosis-related proteins, and (**D**) Quantitative analysis of relative protein expression normalized to β-actin. Data are presented as mean ± SE from three identical experiments. ^###^ *p* < 0.001 denotes significant differences compared to the control; ** *p* < 0.01 and *** *p* < 0.001 compared to treatment with TGF-β1 only.

The TGF-β1-induced apoptosis of the hepatocytes was evaluated by conducting flow cytometry analysis. As shown in Figure 4A,B, the treatment with DIM significantly suppressed the cell death induced by TGF-β1 in a dose-dependent manner compared to the cells treated with TGF-β1 alone. Furthermore, Western blot revealed that the DIM treatment remarkably reduced the protein expression levels of pro-apoptotic markers such as cleaved caspase-3,9, cleaved-Parp, and Bax but increased the expression levels of anti-apoptotic markers such as Bcl2 as compared with treatment with TGF-β1 only, as shown in Figure 4C,D.

### 2.5. DIM Suppresses TGF-β1-Induced Hepatocytes Apoptosis in AML12 Cells

**Figure 5:**
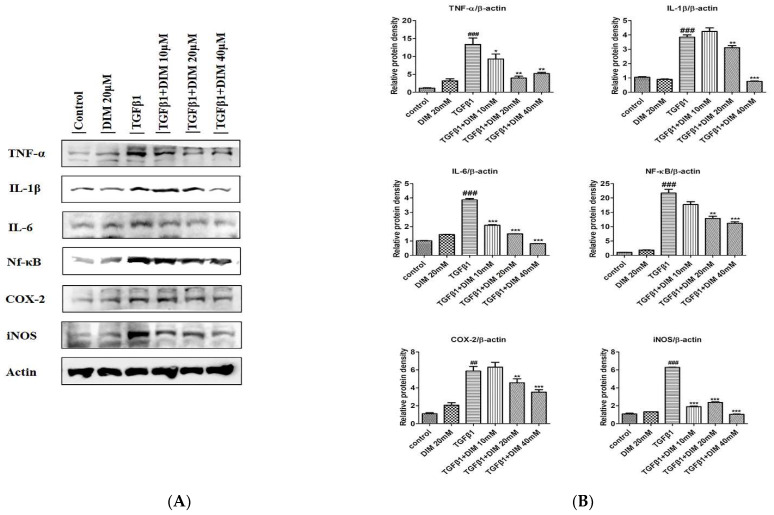
Anti-inflammatory effects of DIM on TGF-β1-induced injury of hepatocytes. (**A**) Western blot analysis revealed anti-inflammatory response of DIM in AML12 cells after TGF-β1 treatment for 24 h. (**B**) Quantification of relative density of inflammatory-related proteins expression normalized to β-actin. Data are mean ± SE of three separate experiments. ^##^ *p <* 0.01; and ^###^ *p* < 0.001 denotes significant differences compared to the control; *, *p* < 0.05; **, *p* < 0.01; and ***, *p* < 0.001 compared to treatment with TGF-β1 only.

TGF-β1 plays a crucial role in the activation of acute and chronic inflammatory responses. It mediates leukocyte recruitment that causes the progression of hepatocyte damage [31]. To determine whether DIM could repress TGF-β1-induced inflammation in AML12 cells, we performed a Western-blot analysis to evaluate levels of inflammation regulatory proteins. As shown in Figure 5A,B, the DIM treatment significantly reduced the expression levels of important cytokines (TNF-α, IL-6, IL-1β) but elevated protein levels of cyclooxygenase (COX-2) and inducible nitric oxide (iNOS) in comparison to the treatment with TGF-β1 only, indicating that DIM might have decreased the TGF-β-induced fibrosis of hepatocytes by repressing inflammatory responses.

### 2.6. DIM Treatment Inhibits Oxidative Stress by Enhancing Antioxidant Activity by Activating the Nrf2/HO-1 Cascade

**Figure 6:**
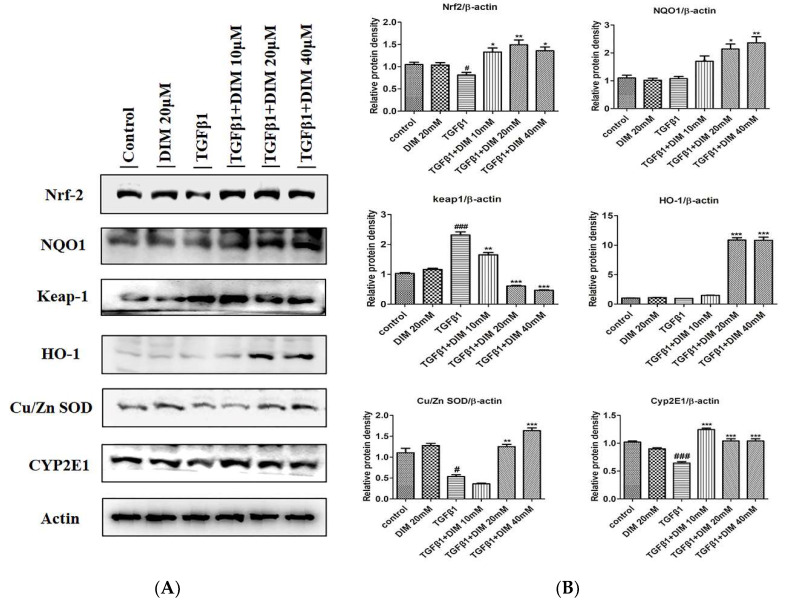
DIM treatment enhances antioxidant ability by activating Nrf2/HO-1 signaling pathway and reducing oxidative stress in TGF-β1-induced hepatocytes injury. (**A**) Protein expression levels of Nrf2, NQO1, Keap-1, HO-1, Cu/Zn SOD, and hepatic cytochrome P450 (CYP2E1) at 24 h after TGF-β1 treatment were determined by Western blot analysis. (**B**) Quantification of proteins expression normalized to β-actin. Data are expressed as mean ± SE of three individual experiments (*n* = 3). ^#^ *p* < 0.05, and ^###^ *p* < 0.001 denotes significant differences compared to the control; * *p* < 0.05, ** *p* < 0.01 and *** *p* < 0.001 compared to treatment with TGF-β1 only.

During liver injury, the activation of the Nrf2/HO-1 cascade can mediate anti-inflammatory and cytoprotective properties. Loss of its activation can induce chronic inflammatory diseases [32]. To evaluate the anti-oxidative mechanism involved in the effect of DIM against TGF-β1-induced oxidative injury to hepatocytes, we measured the protein expression levels of Cu/Zn SOD and Nrf2 and its target proteins by Western blot analysis. As shown in Figure 6A,B, the DIM treatment remarkably restored the protein levels of Nrf2, HO-1, and Cu/Zn SOD, whereas it inhibited the expression levels of Keap-1 and Cyp2E1 in comparison to the treatment with TGF-β1 only.

### 2.7. DIM Attenuates CCl_4_-Induced Chronic Liver Injury

**Figure 7:**
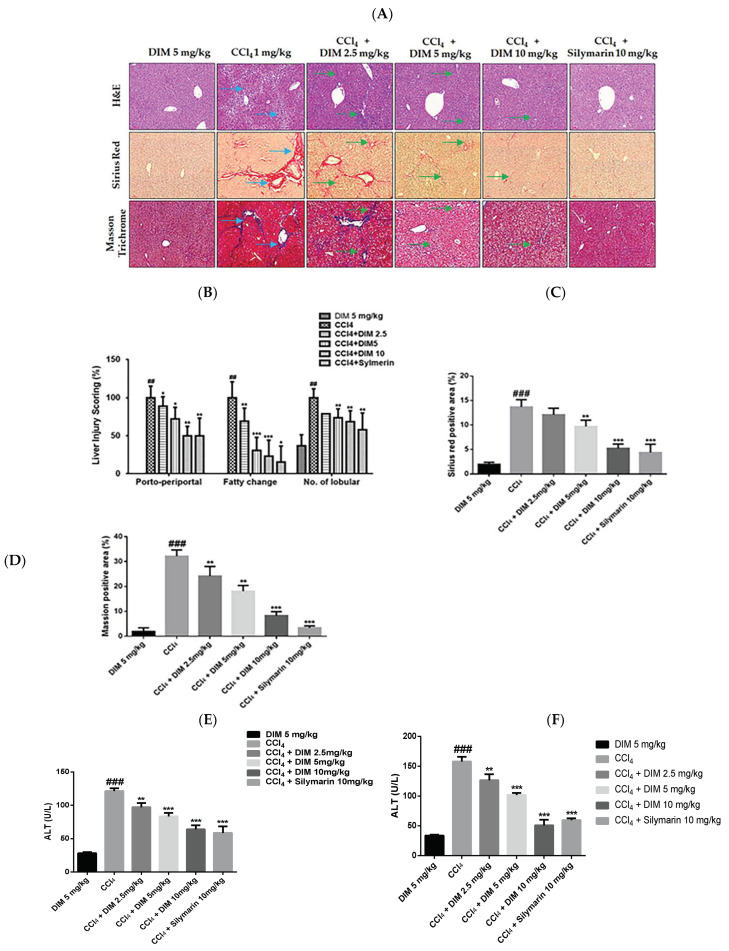
DIM ameliorates CCl_4_-induced mouse liver fibrosis. (**A**) Histopathological damage and fibrosis levels examined by H&E, Sirius Red, and Masson staining of liver; blue arrow indicates the levels of injury after CCl_4_ treatment, and green arrow indicates the recovery after DIM treatment. Observed at 100× magnification. Scale bar: 100 µm. (**B**) Quantitative measurement (%) scoring of liver injury. (**C**,**D**) Statistics (%) of Sirius and Masson positive areas. Serum levels of alanine aminotransferase (ALT, (**E**)) and aspartate aminotransferase (AST, (**F**)) All data are presents as mean ± SD (*n* = 7). ^##^ *p <* 0.01, and ^###^ *p* < 0.001 denotes significant differences compared to the group treated with DIM only. * *p* < 0.05, ** *p* < 0.01, and *** *p* < 0.001 denote significant difference compared to the group treated with CCl_4_.

The CCl_4_-induced chronic liver injury model is one of the most commonly used experimental models to study liver fibrosis [24]. Histopathological damage and fibrosis levels were examined by H&E, Sirius red, and Masson staining. As shown in Figure 7A–D, DIM remarkably attenuated the degree of liver pathology as it reduced necrosis, inflammatory cells, and collagen deposition. In addition, a biochemical analysis of the serum was performed to determine the efficacy of DIM against CCl_4_-induced chronic liver injury. As shown in Figure 7E,F, DIM significantly decreased the serum levels of alanine transaminase (ALT)/aspartate transaminase (AST) and the total bilirubin levels, whereas it restored levels of serum albumin compared to the treatment with CCl_4_ only.

### 2.8. Effects of DIM on Expression Levels of EMT and TGF-β1/Smad in Mouse Liver

**Figure 8:**
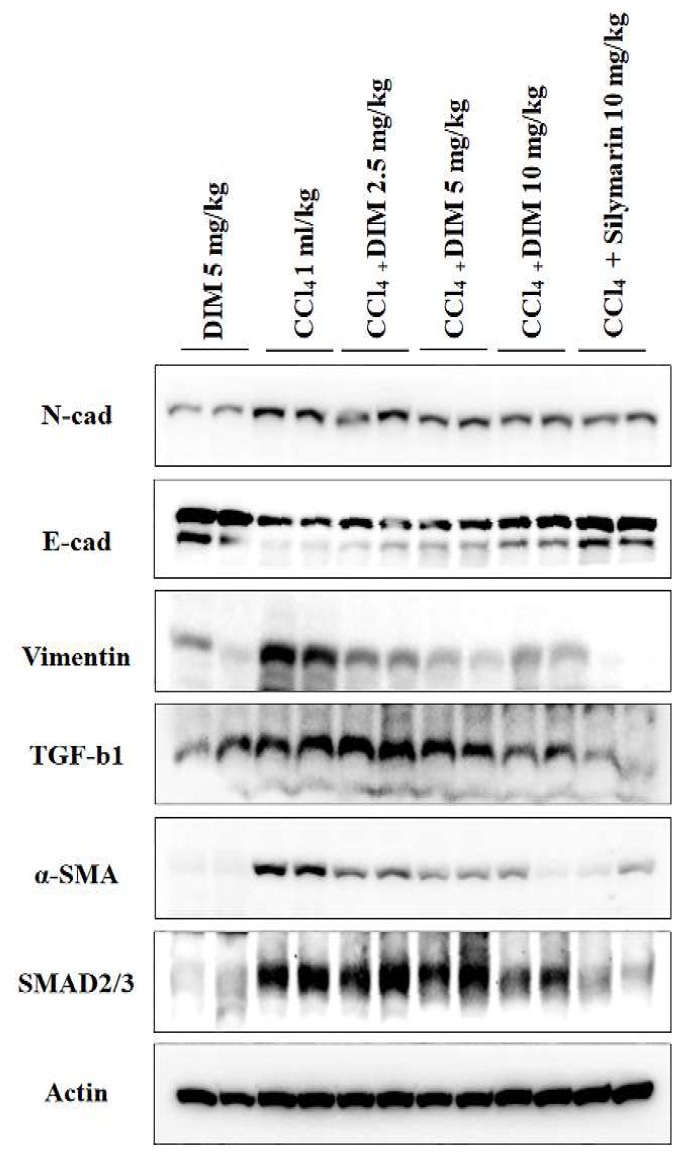
DIM attenuates CCl_4_-induced activation of EMT markers in mouse liver fibrosis. Protein expression levels of EMT markers (α-SMA, TGF-β1, and Smad 2/3) in liver were determined by immunoblotting.

Consistent with our in vitro findings, DIM significantly reversed alternations of CCl_4_-induced EMT dose-dependently in mouse livers, as shown in Figure 8. Immunoblot also revealed that DIM remarkably inhibited the expression of TGF-β1 and Smad 2/3 compared to the treatment with CCl_4_ only group, as shown in Figure 8. These results suggest that the hepatic EMT and TGF-β1/Smad cascade can be regulated by DIM both in vitro and in vivo.

### 2.9. DIM Alleviates Oxidative Stress by Modulating Nrf2 Cascade in CCl_4_-Induced Liver Fibrosis

**Figure 9:**
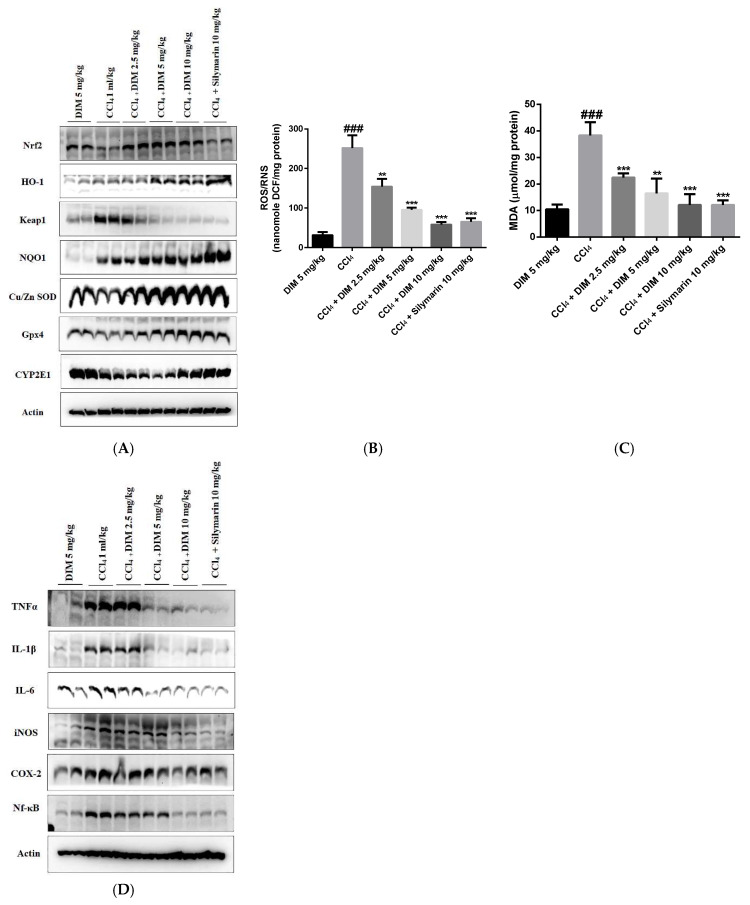
DIM activates hepatic Nrf2 and attenuates CCl_4_-induced ROS, oxidative stress, and inflammatory cytokines in mouse liver fibrosis. (**A**) Expression levels of Nrf2 and antioxidative proteins in liver tissues were determined by Western blotting. (**B**) Effects of DIM on the production of ROS by calculating relative fluorescence unit (RFU)/µg protein. (**C**) Analysis of CCl_4_-induced lipid peroxidation by measuring MDA levels using a commercial kit. (**D**) Anti-inflammatory effects of DIM on the expression of inflammatory cytokines by Western blotting. All data are presented as mean ± SD (*n* = 7). ^###^ *p* < 0.001 denotes significant differences compared to the group treated with DIM only; ** *p* < 0.01 and *** *p* < 0.001 denote significant difference compared to the CCl_4_ group.

Oxidative stress and inflammatory responses are generally used as markers in the pathogenesis of liver fibrosis. As shown in Figure 9A, the DIM treatment promoted the hepatic nuclear translocation of Nrf2 and increased the expression of downstream antioxidative proteins along with GPx-4 compared to the treatment with CCl_4_ only. Similarly, we examined ROS and oxidative stress-related molecules in mouse liver. As shown in Figure 9B,C, the DIM treatment significantly reduced MDA content and ROS fluorescence intensity. We next examined the levels of the inflammatory response induced by CCl_4_. As shown in Figure 9D, DIM significantly inhibited the expression of inflammatory cytokines (TNF-α, IL-6, IL-1β) and suppressed levels of two inducible enzymes (COX-2 and iNOS), suggesting that DIM could protect cells against chronic liver injury by alleviating oxidative stress and inflammatory responses.

## 3. Discussion

Liver fibrosis is a type of chronic liver disease that can cause irreversible damage to the liver due to the accumulation of ECM proteins [8]. It precedes the progression of liver cirrhosis, liver failure, and hepatocellular carcinoma, which is an end stage of chronic liver damage [33]. The treatment of liver disease with conventional and synthetic drugs has been controversial due to poor management and serious side effects. Therefore, it is necessary to discover traditional and herbal medicine with potential therapeutic effects on liver diseases. 3,3′-diindolylmethane (DIM) is a plant-derived natural flavanol extracted from cruciferous vegetables such as broccoli, Brussels sprout, cabbage, cauliflower, and kale. We have previously reported that DIM might possess anti-cancer, anti-inflammatory, and antioxidant properties [27,28]. However, DIM’s anti-fibrotic effect and its molecular mechanism in experimental liver fibrosis are yet to be determined. In the present study, we established in vitro and in vivo models of chronic liver injury and evaluated the possible mechanism involved in the ability of DIM to alleviate CCl_4_-induced liver fibrosis in mice and TGF-β1 mediated EMT in AML12 cells.

Considering the pleiotropic effects of TGF-β1 on cell proliferation, death, and division of various forms of liver cells, its capacity to induce EMT in epithelial cells or end EMT in endothelial cells and its potential to function as an immune modulator requires a deeper understanding to develop an effective therapy for targeting the TGF-β1 pathway in liver diseases considering its complex role in the liver [34,35]. During fibrogenesis, TGF-β1 signaling can modulate a broad spectrum of cellular events. It is involved in collagen synthesis and the progression of chronic liver diseases [36]. A previous study has reported that TGF-β1 is significantly induced in liver fibrosis and cirrhosis. Therefore, it is crucial to investigate TGF-β1-induced EMT and apoptosis in mouse hepatocytes to comprehend the mechanism underlying the progression of liver cirrhosis [11]. CCl_4_ is a well-known hepatotoxin used to prepare a rodent model of liver fibrosis that partially resembles important properties of human liver fibrosis [37]. In the liver, CCl_4_ can produce free radical trichloromethyl by metabolizing cytochrome P450 enzymes, resulting in lipid peroxidation and hepatic injury [38]. Recently, we have shown the protective effects of DIM against CCl_4_-induced acute liver injury [27]. Based on our previous study, we further evaluated the implication of DIM in chronic liver injury.

Epithelial mesenchymal transition (EMT) is a process by which epithelial cells transform into mesenchymal stem cells due to loss of their cell polarity, loss of cell-cell adhesion, remodeling of cytoskeleton, and increased invasion and migratory abilities [39]. EMT can also increase collagen synthesis from hepatocytes and reduce albumins [40]. Numerous studies have explored the mechanism involved in the effect of EMT on the progression of hepatic fibrogenesis in chronic liver injury and suggested that EMT might be a potential therapeutic target for developing anti-fibrogenic strategies [41,42]. Previous studies have shown that TGF-β1-induced EMT in AML12 hepatocytes can result in the progression of liver fibrogenesis through the TGF-β/Smad3 signaling pathway [43], which is characterized by the reduced expression of epithelial marker E-cadherin, ZO-1, and the increased expression of mesenchymal proteins such as α-SMA, fibronectin, N-cadherin, collagen I, and vimentin [44,45]. In the present study, the DIM treatment restored the expression of E-cadherin and ZO-1 but reduced the protein levels of vimentin, N-cadherin, α-SMA, Smad2/3, and Snail in TGF-β1-treated AML-12 cells. Furthermore, numerous studies have suggested that EMT and TGF-β signaling can suppress the progression of CCl_4_-induced liver fibrosis [46]. On the other hand, an increasing number of studies have revealed that mesenchymal markers are increased in primary hepatocytes isolated from a rat model of liver fibrosis [47]. Similarly, a series of natural products can reduce the EMT of hepatocytes and alleviate mouse liver fibrosis provoked by CCl_4_ [46,48]. In line with this study and our in vitro findings, DIM significantly repressed EMT in mouse livers and restored expression levels of E-cadherin reduced by CCl_4_, whereas it inhibited the expression levels of mesenchymal proteins such as α-SMA, N-cadherin, and vimentin compared to the treatment by CCl_4_ only. Furthermore, the activation of the TGF-β/Smad3 cascade enhanced the progression of liver fibrosis [49]. A recent study has shown increased hepatic canonical activation of Smad2/3 in an experimental model of liver fibrosis induced by CCl_4_ and thioacetamide [50]. In line with the recent study, we found that the TGF-β/Smad3 signaling pathway was involved in the protection of DIM against liver fibrosis. Therefore, we evaluated levels of TGF-β1 and Smad 2/3 by immunoblotting. The results showed that DIM could inhibit the expression of CCl_4_-induced TGF-β1 and Smad 2/3, suggesting that DIM could suppress the progression of CCl_4_-induced liver fibrosis by inhibiting the TGF-β1 and Smad3 signaling pathways.

Apoptosis is a programmed cell death process. It is important for the elimination of dysfunctional and damaged cells to maintain homeostasis [51,52]. Several studies have shown the key role of TGF-β in regulating apoptosis [53,54]. At the same time, others have suggested that TGF-β can induce oxidative stress that can activate H_2_O_2_-generating NADH oxidase, which is known to causes the overproduction of intracellular ROS in various cell types [53,55]. In addition, the treatment of hepatocytes with TGF-β not only induces ROS but also suppresses levels of anti-oxidative enzymes such as superoxide, glutathione peroxidase (GPx), and catalase [56,57]. In addition, ROS can induce mitochondrial membrane potential permeabilization both in vitro and in vivo [58]. However, there is no strong evidence on the mechanism by which this cytokine induces cell death. In the present study, we demonstrated the involvement of mitochondria in the TGF-β1-induced apoptosis of hepatocytes. Mitochondria are major producers of ROS. They are also the main target of ROS. In mitochondria, over-production of ROS and free radicals contribute to elevated expression of antioxidant enzymes such as Mn-SOD and Cu/Zn-SOD to prevent mitochondrial oxidative damage [59]. The loss of mitochondrial membrane depolarization and the loss of mitochondrial membrane potential (MMP) resulting from the overproduction of ROS are the first steps of the mitochondria-mediated intrinsic apoptosis pathway [60,61]. In the past, several studies have suggested that treatment with TGF-β1 can induce ROS production and result in the loss of mitochondrial membrane potential, releases cytochrome c, and the activation of caspase-3, leading to the initiation of mitochondrial-dependent apoptosis [62,63]. In the present study, the DIM treatment significantly suppressed the TGF-β1-induced apoptosis of AML-12 hepatocytes by reducing ROS overproduction and the loss of MMP, suggesting that DIM could protect AML-12 cells against oxidative stress-induced mitochondrial dysfunction and apoptosis.

To gain more insight into the potential essential mechanism of the hepatoprotective effect of DIM, we examined the effects of DIM on the regulation of Nrf2/HO-1 signaling. The Nrf2/ARE pathway is one of the most essential defense mechanisms in the protection of the liver against toxic chemicals and their potentially damaging metabolites [64]. Nrf2 is a key transcription factor that can regulate cellular antioxidant response elements (AREs) responsible for the expression of their battery of genes such as HO-1, GST, GCLC, and GCLM [65]. It is primarily regulated by Kelch-like ECH-associated protein (Keap1) [66]. The antioxidant ability of the liver is increased when Nrf2 separates from Keap1 during oxidative stress, leading to the synthesis of antioxidize and phase II detoxification enzymes, which can prevent liver fibrosis [67,68]. In this study, a TGF-β-induced liver disease model with DIM as an intervention was used to observe expression levels of Nrf2 and its downstream products NQO1 and HO-1 after treatment with different concentrations of DIM. Our results showed that expression levels of Nrf2 and its downstream NQO1 HO-1 and Keap1 were significantly increased in the DIM treated group than in the TGF-β1 group. Several studies have suggested that liver fibrosis is strongly aggravated due to the enhanced inflammation in Nrf2 knockout mice during long-term CCl_4_ exposure [69]. Liver fibrosis is usually caused by the lack of antioxidant defenses in clinical and experimental contexts of liver fibrosis. CCl_4_ intoxication and bile duct ligation are commonly used to determine the role of oxidative stress in experimental models of fibrosis [70]. In line with this study and our in vitro findings, DIM activated the Nrf2 cascade and improved the oxidative parameters. It also attenuated MDA levels in mouse livers, suggesting that regulation of Nrf2 cascade and improvement of antioxidant ability might make a pivotal contribution to the hepatoprotective effect of DIM. In addition to oxidative stress, the inflammatory cascade also plays a key role in the progression of liver fibrosis in response to hepatocyte damage and death [71]. In our study, TGF-β1 increased the expression levels of inflammatory cytokines in the AML12 cells. Such increases were inhibited by the DIM treatment. Recently, we have shown that DIM possesses an anti-inflammatory ability to inhibit the inflammatory response in CCl_4_-induced acute liver injury [27]. Consistent with our previous study and the in vitro findings of the present study, we found that DIM significantly reduced the expression levels of IL-1β, IL-6, COX-2, iNOS, and TNF-α in CCl_4_-induced liver fibrosis. These results suggest that the anti-fibrotic ability of DIM may be associated with its ability to inhibit oxidative stress and reduce pro-inflammatory cytokines in CCl_4_-induced chronic liver injury.

Serum biochemical indexes and liver histopathological changes are important parameters to determine hepatoxicity in an experimental model of liver fibrosis. Previously, we have found that DIM apparently improved pathological lesions of liver injury in an acute liver injury model by attenuating oxidative stress, inflammation, and apoptosis [27]. Consistently with this study, our present findings also revealed that DIM significantly reduced serum AST and ALT levels compared to the model group. A previous study suggested that increased serum ALT and total bilirubin levels intimate liver damage, whereas decreased albumin levels in chronic liver damage could impair liver cell protein synthesis [72]. In the current study, the DIM treatment suppressed levels of total bilirubin but restored albumin levels compared to the model group (treatment with CCl_4_ only). Histopathological studies of liver sections in this study also confirmed the degree of fibrosis by measuring collagen deposition in liver tissues. The results from the Sirius red and Masson staining revealed that the DIM treatment remarkably ameliorated CCl_4_-induced hepatic fibrogenesis.

## 4. Materials and Methods

### 4.1. Chemicals and Reagents

DIM (purity ≥ 98%), CCl_4_, Masson Trichome, and Sirius red (Direct red 80) were bought from Sigma-Aldrich Chemical Co. (St. Louis, MO, USA). Recombinant transforming growth factor-beta 1 (TGF-β1) was purchased from R&D system (Minneapolis, MN, USA). Dulbecco’s Modified Eagle’s Medium (DMEM/F12), Fetal Bovine Serum (FBS), and penicillin–streptomycin solution were purchased from Hyclone (Logan, UT, USA). Insulin-transferrin-selenium (ITS) premix was purchased from Corning Inc. (Bedford, MA, USA).

### 4.2. Cell Cultures and Drug Treatment

The AML-12 cells (ATCC, VA, USA) were cultured in DMEM/F12 medium supplemented with 10% FBS and 1% penicillin–streptomycin antibiotics along with 5 µg/mL ITS premix and 50 ng/mL dexamethasone at 37 °C in a humidified condition with 5% CO_2_. The cell line was authenticated regularly using A Short Tandem Repeats (STR) ATCC panel, monitored for mycoplasma contamination, and tested negative for mycoplasma contamination. To determine the effect of DIM on AML12 cell viability, the cells were seeded into 96-well plates at a cell density of 5 × 10^3^ cells and cultured for 24 h. These cells were treated with recombinant TGF-β1 5 ng/mL together with DIM (0, 10, 20, 40, 60 µM) for 24, 48, and 72 h in serum-free media. After the media was removed, the cells were incubated with 50 µL of 5 mg/mL 2,5-diphenyl tetrazolium bromide (MTT; Sigma Aldrich, St. Louis, MO, USA) for 2–4 h. Transformed purple formazan crystals were solubilized in dimethyl sulfoxide (DMSO), and the optical density was measured at a wavelength of 575 nm.

### 4.3. Experimental Animals

Healthy male C57BL6 mice at 8 weeks old weighing 19–22 g were purchased from Koatech (Pyeongtake, Korea). All of the mice were housed under appropriate conditions and provided with free access to water and food. Every experiment in this study was conducted according to the animal ethical guidelines. The study protocols were approved by the Institutional Animal Care and Use Committee of Jeonbuk National University, Jeonju, South Korea (Approved no: CBNU 2020-049).

### 4.4. Experimental Model and Drug Treatment

C57BL6 mice were randomly divided into 6 groups (7 mice per group) as follows:

(I) DIM group: Mice were treated with 5 mg/kg DIM subcutaneously daily for 4 weeks.
(II) CCl_4_ group: Mice were treated with 10% CCl_4_ solution in mineral oil intraperitoneally with a dose of 1 mL/kg for three consecutive days for 8 weeks.
(III) CCl_4_ + DIM 2.5 mg/kg group: Mice were i.p. injected with CCl_4_ for the first 4 weeks, followed by subcutaneous administration of DIM at 2.5 mg/kg for the last 4 weeks together with CCl_4_.
(IV) CCl_4_ + DIM 5 mg/kg group: Mice were i.p. injected with CCl_4_ for the first 4 weeks, followed by subcutaneous administration of DIM at 5 mg/kg for the last 4 weeks together with CCl_4_.
(V) CCl_4_ + DIM 10 mg/kg group: Mice were i.p. injected with CCl_4_ for the first 4 weeks, followed by subcutaneous administration of DIM at 10 mg/kg for the last 4 weeks together with CCl_4_.
(VI) CCl_4_ + Silymarin 10 mg/kg group: Mice were i.p. injected with CCl_4_ for the first 4 weeks, followed by subcutaneous administration of DIM at 10 mg/kg for the last 4 weeks together with CCl_4_.

### 4.5. FACS Analysis

Annexin V/PI staining was used to determine early and late apoptosis of the AML-12 hepatocytes. Briefly, the AML-12 cells were seeded into a 60-mm culture dish at a cell density of 5 × 10^4^ cells overnight and then incubated with a culture medium containing TGF-β1 at 5 ng/mL and DIM at 0, 10, 20, or 40 µM for 24 h. The cells were washed with PBS twice and trypsinized. The cells were then collected and centrifuged at 1500 rpm for 3 min at 15 °C. An Annexin V-FITC apoptosis kit (Trevigen Inc., Gaithersburg, MD, USA) was then used to determine apoptosis by flow cytometry, according to the manufacturer’s instructions.

### 4.6. Detection of Reactive Oxygen Species (ROS)

To determine the intracellular ROS levels, the AML-12 cells were stained with a fluorescent dihydroethidium (DHE) probe (Invitrogen, Carlsbad, CA, USA). In this process, intracellular DHE is oxidized to ethidium, which binds to DNA and stains the nuclei a bright red fluorescent color. The AML-12 cells were cultured in glass-bottom confocal dishes at a cell density of 5 × 10^3^ cells and treated with recombinant TGF-β1 and DIM, as described previously [28]. The cells were washed with warm PBS twice and then fixed with ice-cold methanol for 10–15 min at room temperature. After fixation, the methanol was discarded, and 5 µM of DHE solution (DHE was diluted in PBS) was applied to the cells, and the plate was covered with an aluminum foil and incubated at 37 °C in the dark for 35 min. The cells were then washed with PBS, labeled with 4′,6-diamidino-2-phenylindole dihydrochloride (DAPI) for 5–10 min, and observed under a fluorescence microscope using an x63 oil immersion objective lens (Axioskop 2 plus, Carls Zeiss, Gottingen, Germany). Furthermore, to determine the amount of ROS production in mice with CCl_4_-induced liver fibrosis, an Oxiselect^TM^ In Vitro ROS/RNS assay kit (Cell Biolabs, Inc., San Diego, CA, USA) was used. The mouse blood serum was scanned with fluorescence of dichlorofluorescein (DFC). The optical density was measured using a SpectraMax Gemini XS fluorimeter (Molecular Devices, Sunnyvale, CA, USA) at an excitation wavelength of 480 nm and an emission wavelength of 530 nm, following the manufacturer’s protocol.

### 4.7. Analysis of Mitochondrial Membrane Potential (ΔΨm)

The changes in mitochondrial membrane potential were determined using a tetramethylrhodamine ethyl ester perchlorate (TMRE) mitochondrial membrane potential assay kit (Abcam) following the manufacturer’s instruction. The AML-12 cells were seeded into 96-well plates at a cell density of 1 × 10^4^ cells per well and cultured for 24 h. These cells were treated with a medium containing recombinant TGF-β1 at 5 ng/mL and DIM at indicated concentrations (0, 10, 20, and 40 µM) for 24 h. The medium was then discarded, and the cells were stained with TMRE (400 nmol/L) for 20 min at room temperature in the dark. After the cells were washed twice with PBS, TMRE intensity was measured at excitation and emission wavelengths of 549 nm and 575 nm, respectively, using a fluorescence plate reader.

### 4.8. Assessment of Biochemical Parameters

Sera were isolated from the blood samples for analyzing alanine transaminase (ALT), aspartate aminotransferase (AST) (Asan Pharm. Co., Ltd., Seoul, Korea), total bilirubin, and albumin (Abnova, Taiwan) levels spectrophotometrically according to each manufacturer’s instructions.

### 4.9. Measurements of Lipid Peroxidation

The amount of MDA in the mouse liver tissue was analyzed spectrophotometrically using a thiobarbituric acid-reactive substances (TBARS) assay kit (Cell Biolabs, Inc., San Diego, CA, USA), as suggested by the manufacturer’s instructions.

### 4.10. Histological Assessment

Paraffin-embedded mouse liver tissue sections (5 µm in thickness) were deparaffinized and dehydrated with xylene and alcohol, respectively. Morphological changes were determined after staining tissues with hematoxylin and eosin (H&E). The degree of fibrosis was determined after staining the tissue with 0.1% Sirius red and Masson’s trichrome stains.

### 4.11. Immunoblotting

The proteins from the AML12 cells and liver tissues were extracted as previously described [27,28]. The changes in the protein expression levels were determined by running proteins in SDS-PAGE gels, electro-transferring them onto PVDF membranes, blocking with 5% skimmed milk, and then incubating with specific primary and horseradish peroxidase-conjugated secondary antibodies. All of the primary and corresponding secondary antibodies used in this study are listed in Table 1. The protein signals were enhanced and detected using a chemiluminescence detection system.

### 4.12. Statistical Analysis

All of the results are expressed as mean ± standard error. The experimental results were obtained after repeating each experiment at least three times. One-way analysis of variance (ANOVA) followed by Dunnett’s test or turkey’s post hoc test in Prism 7 software (Graph Pad Software, Inc., San Diego, CA, USA) was used to evaluate statistical significance. A *p*-value of less than 0.05 (*p* < 0.05) was considered statistically significant.

## 5. Conclusions

**Figure 10:**
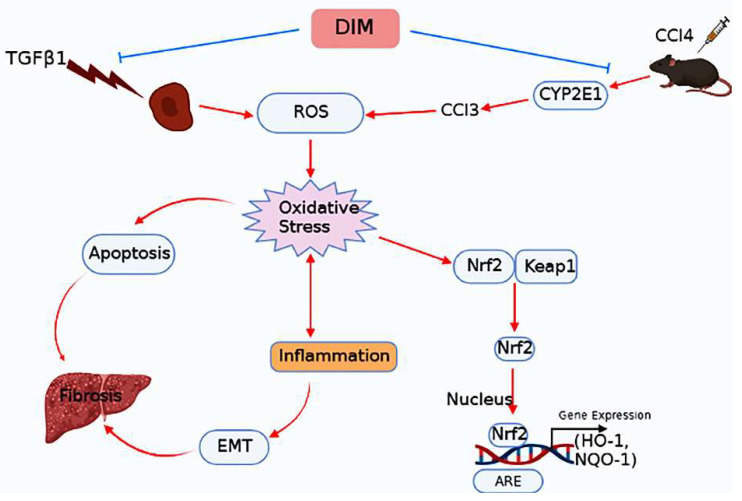
A schematic diagram showing molecular mechanisms involved in the protective effect of DIM against chronic liver injury by modulating ROS, oxidative stress, EMT, and fibrosis. In the figure, DIM inhibits TGF-β1-induced EMT by inhibiting intracellular ROS, oxidative stress, and apoptosis of heptocytes to prevent fibrosis, whereas DIM protects mice against CCl_4_-induced chronic liver injury by inhibiting ROS-mediated oxidative stress and Nrf2 cascade activation, thus reducing inflammation, EMT, and fibrosis.

Taken together, our results revealed that DIM suppressed TGF-β1-induced EMT in AML-12 cells by regulating the Nrf2/HO-1 cascade. Briefly, DIM elicited nuclear translocation of Nrf2 and induced the transcription of Nrf2-responsive antioxidative genes. In addition, DIM inhibited the loss of mitochondrial membrane potential by reducing the production of intracellular ROS that resulted in suppression of TGF-β1-induced hepatocytes apoptosis. Furthermore, results from our in vivo study revealed that DIM attenuated CCl_4_-induced mouse liver fibrosis and modulated levels of hepatic EMT markers. In mice livers, DIM enhanced Nrf2 levels and suppressed Smad2/3 expression (Figure 10). Altogether, our study concludes that DIM could be used as a new potential therapeutic agent for inhibiting liver fibrosis.

## Figures and Tables

**Table 1:** List of specific antibodies and secondary antibodies used for immunoblotting.

S.N	Target	Blocking Solution	Dilution	Secondary	Manufacturer	Catalogue Number
1	β-actin	5% Skim Milk	1:3000	Mouse IgG	Sigma Aldrich	A5441
2	COX-2	%5 BSA	1:1000	Rabbit IgG	Cell Signalling	#12282
3	TGF-β1	%5 BSA	1:2000	Rabbit IgG	Abcam	Ab66043
4	E-cadherin	%5 BSA	1:2000	Rabbit IgG	Cell Signalling	#3195
5	C-Caspase-3	%5 BSA	1:1000	Rabbit IgG	Cell Signalling	#9661
6	Caspase-9	%5 BSA	1:1000	Mouse IgG	Cell Signalling	#9508
7	C-PARP	%5 BSA	1:1000	Rabbit IgG	Cell Signalling	#13669
8	N-cadherin	%5 BSA	1:2000	Mouse IgG	Abcam	Ab18203
9	Keap-1	5% Skim Milk	1:1000	Rabbit IgG	Santa Cruz	Sc-33569
10	α-SMA	5% Skim Milk	1:2000	Mouse IgG	Sigma	A2547
11	Nrf-2	5% Skim Milk	1:1000	Rabbit IgG	Santa Cruz	sc-81342
12	Nf-κB	5% Skim Milk	1:3000	Rabbit IgG	Santa Cruz	sc-8008
13	GpX4	%5 BSA	1:1000	Mouse IgG	Santa Cruz	Sc-166570
14	HO-1	%5 BSA	1:1500	Mouse IgG	Enzo Life Science	136960
15	ZO-1	%5 BSA	1:1000	Rabbit IgG	Cell Signalling	#13663
16	iNOS	%5 BSA	1:1500	Rabbit IgG	Enzo Life Science	ADI-KAS-NO001
17	IL-1β	5% Skim Milk	1:1000	Rabbit IgG	Santa Cruz	sc-7884
18	IL-6	5% Skim Milk	1:1000	Rabbit IgG	Santa Cruz	sc-57315
19	Bax	5% Skim Milk	1:3000	Rabbit IgG	Santa Cruz	sc-7480
20	Bcl2	5% Skim Milk	1:3000	Rabbit IgG	Santa Cruz	sc-7382
21	Snail	%5 BSA	1:1000	Rabbit IgG	Cell Signalling	#3879
22	PARP	5% Skim Milk	1:2000	Mouse IgG	Santa Cruz	sc-8007
23	Caspase-3	%5 BSA	1:1000	Rabbit IgG	Cell Signalling	#9661
24	TNF-α	%5 BSA	1:1000	Rabbit IgG	Cell Signalling	#12744
25	CYP2E1	%5 BSA	1:1500	Rabbit IgG	Abcam	Ab28146
26	Cu/Zn SOD	%5 BSA	1:3000	Rabbit IgG	Enzo Life Science	ADI-SOD-100
27	NQO1	5% Skim Milk	1:1500	Mouse IgG	Santa Cruz	sc-32793
28	Vimentin	%5 BSA	1:1000	Rabbit IgG	Cell Signalling	#5741
29	Slug	%5 BSA	1:1000	Rabbit IgG	Cell Signalling	#9585

## Data Availability

Not applicable.
